# CD44 expression in curettage and postoperative specimens of endometrial cancer

**DOI:** 10.1007/s00404-014-3407-1

**Published:** 2014-08-17

**Authors:** Michał Wojciechowski, Tomasz Krawczyk, Janusz Śmigielski, Andrzej Malinowski

**Affiliations:** 1 Department of Surgical,Endoscopic and Oncologic Gynecology, Polish Mothers’ Memorial Hospital–Research Institute, 281/289 Rzgowska Street, Lodz, 93-338 Poland; 2 Department of Surgical and Endoscopic Gynecology, Medical University of Lodz, Lodz, Poland; 3 Department of Pathology, Polish Mothers’ Memorial Hospital–Research Institute, Lodz, Poland; 4 Department of Medical Informatics and Statistics, Medical University of Lodz, Lodz, Poland

**Keywords:** Endometrial cancer, Adhesive molecules, CD44, Metastasis

## Abstract

**Background:**

Adhesive molecules like CD44 are well defined key players in the metastatic cascade in many cancers, including endometrial cancer. They could play a role of markers of invasion, metastasis and prognostic factors.

**Aim of the study:**

The aim of the study is to assess a possible role of the CD44 as a marker of invasion in endometrial cancer, both at the moment of preoperative workup and final staging.

**Materials and methods:**

Available for analysis were archival specimens of 51 patients who had underwent curettage and surgery between 2002 and 2007. An immunohistochemical study for CD44 expression was performed in curettage and postoperative specimens. Normal endometrium of 20 randomly chosen patients was used as a control group.

**Results:**

In endometrial cancer the expression of CD44 was significantly more intensive than in normal endometrium. In postoperative specimens, the CD44 expression was weaker in serous than in endometrioid cancer. There was no significant correlation between the adhesion molecule expression and clinicopathological features: grade,depth of invasion, cervical involvement, serosal and adnexal involvement, lymph-vascular space involvement, lymph node and distant metastases nor FIGO stage.

**Conclusions:**

An increased expression of CD44 in endometrial cancer suggests its possible role in pathogenesis of this disease, however, it doesn’t seem to be crucial. Different expression of the CD44 in endometrioid and papillary-serous type may reflect different pathogenesis of these types of cancer. No statistically proved relation between the investigated molecule expression and clinicopathological parameters suggests scepticism about its use in diagnostic process of endometrial cancer.

## Introduction

Endometrial cancer (EC) is currently the fourth most common cancer in women in developed countries and the most common among cancers of the female reproductive tract [1–5]. It is a disease of the elderly; over 80 % of patients are postmenopausal. Therefore, diagnosis of the first symptoms—usually abnormal uterine bleeding—is relatively easy. As a result, most cases are diagnosed at an early stage, when the treatment of choice is surgery [6–8]. In some women, however, lymph node metastases develop, which, if undiscovered, lead to a relapse. Thus, patients at high risk of nodal involvement should be properly identified, accurately staged pending lymphadenectomies, and subsequently treated with individualized adjuvant therapy [9–11]. Unfortunately, contemporary diagnostic modalities are insufficient in finding or predicting lymph node metastases. An intense ongoing debate exists over the indications for lymph node dissection in EC patients. If performed routinely, lymphadenectomy seems an unnecessary overtreatment for many patients with early-stage disease and low risk of lymph node involvement [9–11]. Development of a new diagnostic test or finding a risk factor that could identify patients at high risk of lymph node metastases more precisely has been widely attempted, but none proved efficient enough to become a routine diagnostic procedure.

Adhesive molecules are cell-surface glycoproteins that are responsible for cell–cell and cell–matrix adhesion and interactions. Such interplay between cells and matrix is crucial for tissue architecture and functioning. Cell adhesion molecules anchor cells to their surroundings, regulate cell mobility, and provide cells with critical sensors towards their environment. They are indispensable for proper tissue identification and differentiation, participation in intercellular communication and signaling, and cell function regulation [12–15]. In healthy tissue, loss of adhesion leads to apoptosis—so-called anoikis, cell dedifferentiation and acquisition of invasive, fibroblast-like morphology and disruption of tissue architecture [13–16]. Several families of cell adhesion receptors exist. CD44 is a separate family of cell adhesion molecules that includes the standard (CD44s) and variant isoforms, which are products of alternative splicing. CD44 was initially thought to be a lymphocyte homing receptor that mediates lymphocyte circulation. CD44 is the primary hyaluronic acid (HA) receptor, and is responsible for cell adhesion to this basic component of extracellular matrix [17–21]. CD44 affects carcinogenesis of many cancers through several mechanisms, notably cell migration and metastasis initiation. However, available data on the role of CD44 in EC are inconsistent.

This study aimed to assess CD44 expression in EC, using both curettage and resected specimens, and to look for a correlation between its expression and clinical and pathologic features of EC. This is also the first assessment of CD44 expression in curettage specimens as a means to identify patients at increased risk of lymph node metastases and recurrence.

## Materials and methods

### Material

CD44 expression was evaluated in 49 patients with EC who underwent both diagnostic curettage and surgical staging in the Department of Surgical and Endoscopic Gynecology, Polish Mother’s Memorial Hospital and Research Institute in Lodz, and in two patients who underwent the same procedure in the Ministry of the Interior and Administration Hospital in Lodz between 2002 and 2007, thus providing specimens from 51 patients with EC for further investigation. All of the patients first underwent routine, diagnostic curettages, and then surgical staging after their diagnoses were posted. Forty-one patients then received pelvic lymphadenectomies. The remaining 10 patients were disqualified from lymph node dissection due to poor status performance, advanced age, obesity or other comorbidities. The tissue samples were routinely fixed in 10 % formalin and embedded in paraffin. The control group consisted of archival, paraffin embedded specimens of normal endometrium from 20 patients who underwent curettage and hysterectomy for benign conditions. One pathologist selected representative samples of EC, both from preoperative (curettage) and postoperative material, from which 4-µm thick, paraffin-embedded specimens were prepared for CD44 immunostaining and for hematoxylin–eosin-stained controls. Histological diagnoses were based upon FIGO (1988) criteria, and included 46 endometrioid and five papillary-serous EC types. Tumor grades were G1 (well differentiated) in 24 patients (47 %), G2 in 21 (38.9 %) patients and G3 (poorly differentiated) in five patients (10.9 %). All the cancer cases were analyzed for clinicopathological factors, including FIGO stage, tumor grade, depth of myometrial invasion, cervical involvement, serosal or adnexal invasion, presence of distant metastases, lymph-vascular space involvement and—in 41 patients—lymph node status.

## Ethical considerations

The research was approved by the Bioethical Committee of The Military Division of the Medical University of Łódź, Poland (No. RNN/25/06/KB). This report contains no identifying patient data.

### Immunohistochemistry

Surgical and curettage specimens were fixed in 10 % buffered formalin and embedded in paraffin. Paraffin blocks for each case were then selected, cut into 4-µm-thick sections and mounted on silanized slides. The slides were deparaffinized in xylene, rehydrated with ethanol and washed in Tris-buffered saline. Epitopes were retrieved at high temperature in Dako Target Retrieval Solution; slides were then cooled and rinsed in distilled water. Peroxidase activity was blocked with 3 % solution of H_2_O_2_ (Dako Peroxidase Block, Dako EnVision). The slides were then incubated with primary antibody: (Dako CD44 mouse monoclonal antibody no. M7082) and then for 30 min with secondary antibody: Dako EnVision K4007 detection system and 3-3′-diaminobenzidine (DAB) as chromogen. The negative control was performed using saline instead of primary antibody. A human tonsil section was used as a positive control.

### Immunohistochemistry staining score

Staining intensity was evaluated with a semiquantitative score. Percentages of CD44^+^ cancer cells in 10 randomly chosen fields of each slide were scored as: 0 points for 0–5 %, 1 point for 5–25 %, 2 for 25–75 % and 3 points for >75 % cells. The average score from the 10 fields was calculated for an immunohistochemical index representing each slide. The intensity of reaction was not subjected to a comparative study.

### Statistics

Non-normal distributions were verified with a Shapiro–Wilk test. U–Mann–Whitney and Kruskal–Wallis tests were used to compare the differences between the two groups. The Spearman test was used to verify the correlation in CD44 expression between curettage and resected specimens. *P* < 0.05 was considered statistically significant.

## Results

Among the 51 patients who underwent surgery for EC, 46 (90.2 %) had endometrioid and 5 (9.8 %) had papillary-serous histopathology. Tumor grades were G1 in 24 patients (47 %) G2 in 21 patients (38.9 %) and G3 in 6 patients (11.8 %). Of the 46 endometrioid cancers, 21 (45.7 %) were G1 cancers, 19 (41.3 %) were G2 and 5 (10.9 %) were G3. Of the 5 papillary-serous tumors, 3 were G1, 1 was G2 and 1 was G3. Six patients (11.8 %) showed no myometrial invasion, 19 (37.3 %) had shallow invasion and 26 (~51 %) had deep invasion; with cervical involvement in 12 cases and serosal or adnexal involvement in another 12 cases (23.5 %). Distant metastases (excluding lymph nodes metastases) were found in 4 patients (7.8 %) and lymph-vascular space involvement (LVSI) in three patients (5.9 %). Ten patients out of 41 (24.4 %) who underwent lymphadenectomy had positive lymph nodes. According to the FIGO 1988 classification, 28 patients (54.9 %) were in the FIGO I stage, 6 (11.8 %) in stage II, 13 (25.5 %) in stage III, and 4 (7.8 %) in stage IV (Table 1).

**Table 1:** The clinicopathological data

The clinicopathological data from surgical staging	*N* = 51 (%)
Histopathology	
Adenocarcinoma endometrioides endometrii	46 (90.2 %)
Adenocarcinoma serosum	5 (9.8 %)
Grading	
G1	24 (47 %)
G2	21 (38.9 %)
G3	6 (11.8 %)
Depth of myometrial invasion	
No	6 (11.8 %)
<50 %	19 (37.3 %)
>50 %	26 (50.9 %)
Involvment of the cervix	
No	39 (76.5 %)
Yes	12 (23.5 %)
Adnexal/serosa involvement	
No	39 (76.5 %)
Yes	12 (23.5 %)
Distant metastases (lymph nodes not included)	
No	47 (92.2 %)
Yes	4 (7.8 %)
Lymph node metastases (in 41 lymphadenectomies)	
No	31 (75.6 %)
Yes	10 (24.4 %)
Lymph-vascular space involvement	
No	48 (94.1 %)
Yes	3 (5.9 %)
FIGO 1988 stage	
I	28 (54.9 %)
II	6 (11.8 %)
III	13 (25.5 %)
IV	4 (7.8 %)

**Fig. 1:**
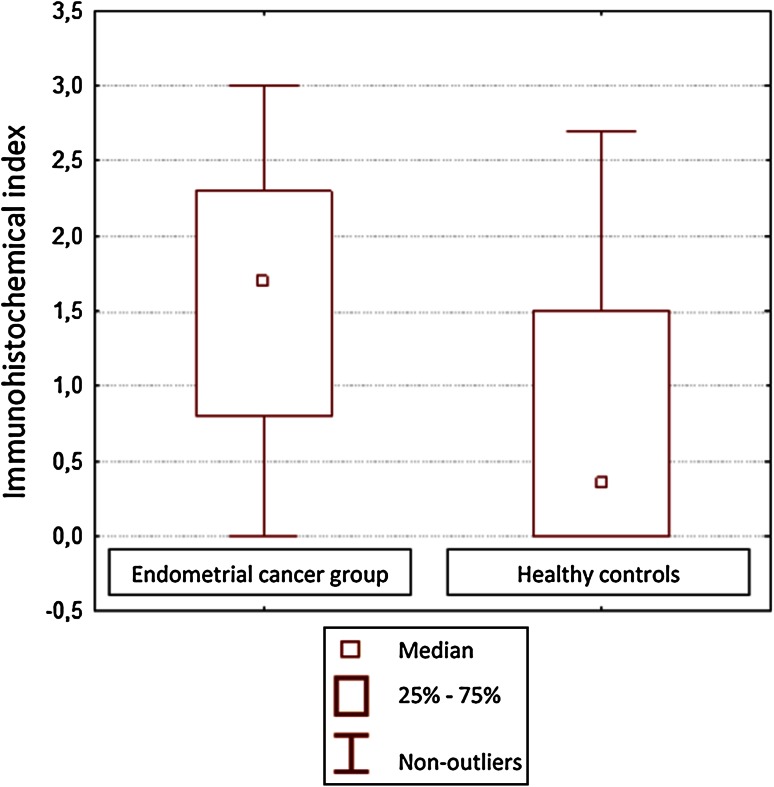
The CD44 expression in normal endometrium and in endometrial cancer in curettage samples (*P* < 0.05)

**Fig. 2:**
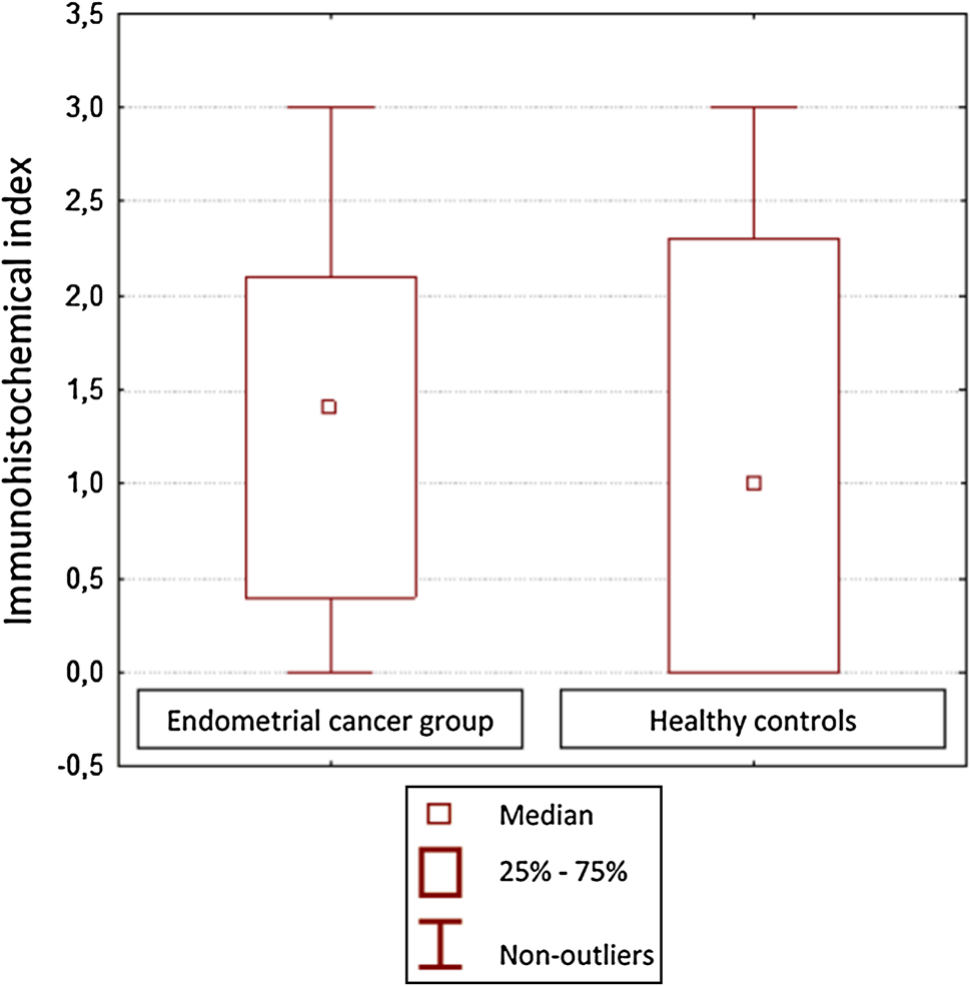
The CD44 expression in normal endometrium and in endometrial cancer in postoperative specimens (*P* > 0.05)

**Fig. 3:**
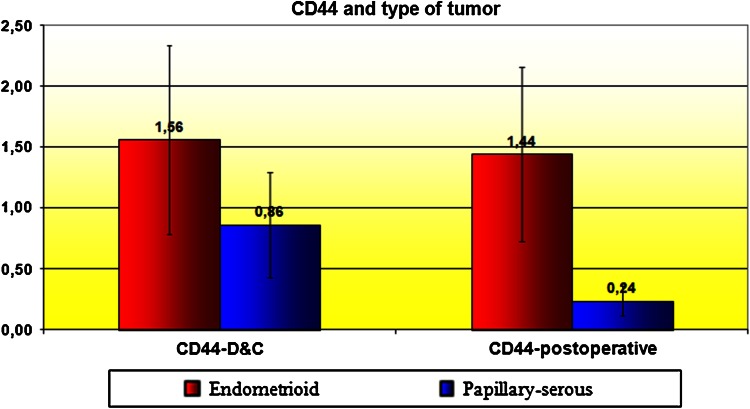
CD44 and type of tumor

**Fig. 4:**
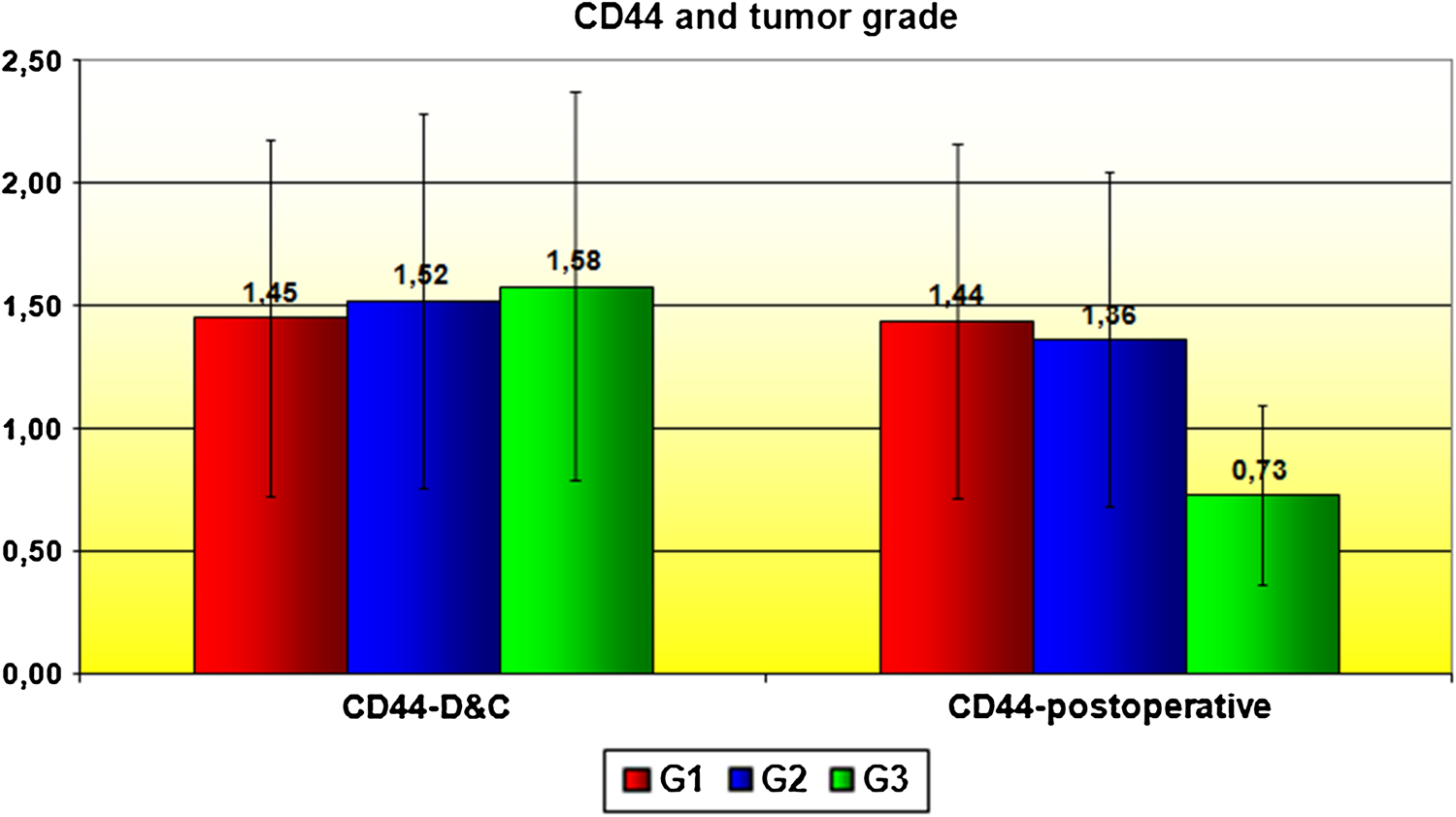
CD44 and tumor grade

**Fig. 5:**
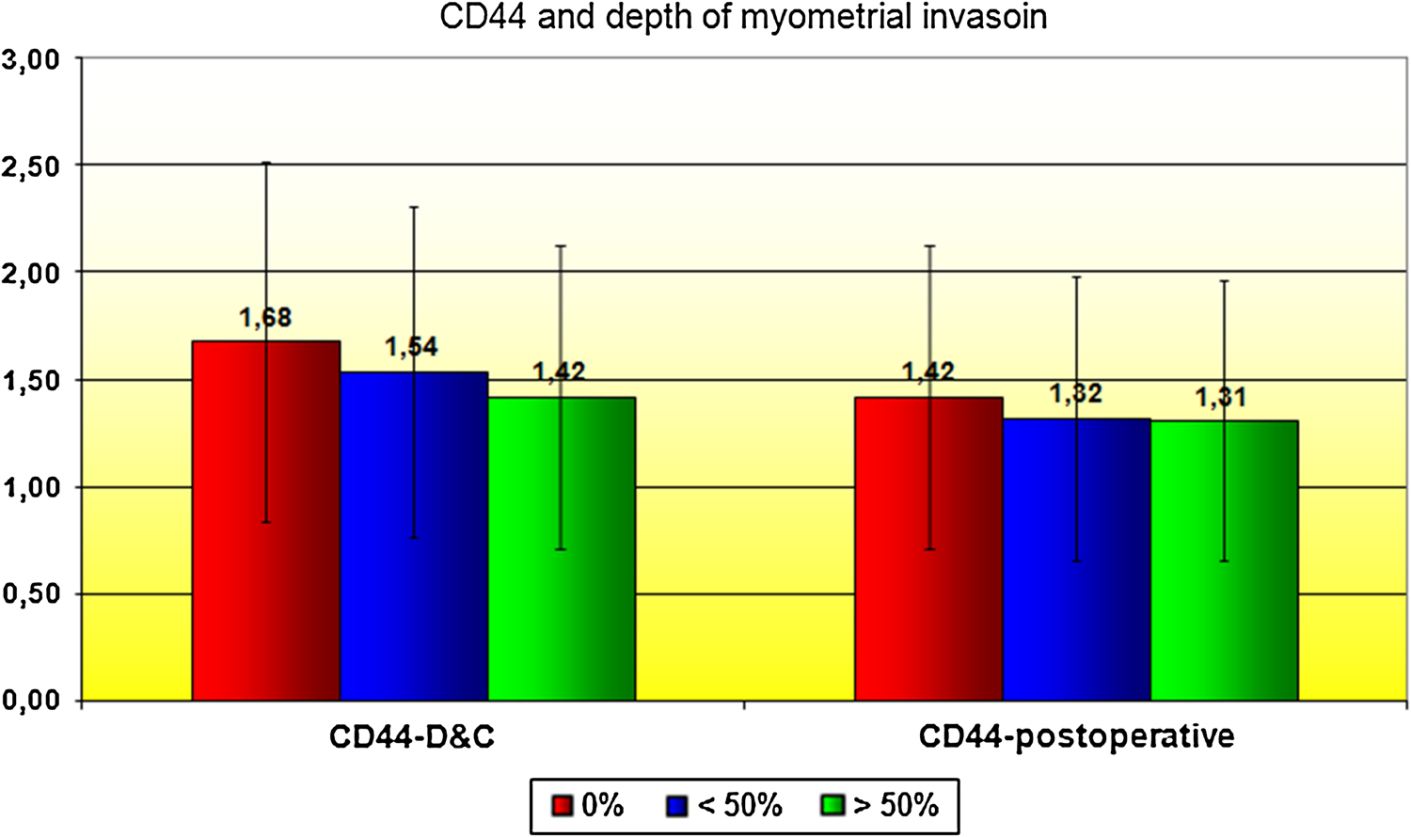
CD44 and depth of myometrial invasoin

Correlation between CD44 expression in curettage and resection specimens was significant for cancerous tissues (*P* = 0.001) and almost significant (*P* = 0.055) in the control group. CD44 expression in EC tissues was stronger than in normal epithelium, but only significantly so for the curettage specimens (*P* < 0.05; Figs. 1, 2). However, CD44 was more expressed in endometrioid than in papillary-serous EC specimens, both from curettage (ns, *P* > 0.05) and hysterectomy (*P* < 0.05) (Fig. 3). CD44 staining intensity did not vary significantly by tumor grade, although it decreased slightly with grade in hysterectomy specimens (Fig. 4). In both curettage and resection specimens, CD44 staining intensity was inversely, but not significantly, correlated, with myometrial invasion depth (*P* > 0.05; Fig. 5).

We found no significant correlation between CD44 expression and lymph node metastases, distant metastases, cervical infiltration, serosal or adnexal involvement, lymph-vascular space involvement or FIGO stage.

## Discussion

Adhesive molecules, including CD44, are cell-surface glycoproteins that affect structural and functional tissue organization. They reportedly affect many cancer processes, particularly cell migration and invasion. Alterations of adhesion intensity can free a cell from its environment and allow it to migrate and become invasive with parallel morphological transformation—a hallmark of carcinogenesis [13–17, 20–24]. Several studies have indicated that CD44 might participate in EC carcinogenesis, but the data are inconsistent. Most studies report altered CD44 expression but vary in expression patterns, often with contradictory results. The variations include under- or over-expression and, most often, expression of variant CD44 forms, particularly CD44v3 and v6. In most reports the CD44 and its variants are expressed more in EC tissue than in normal endometrium [25–36]. This study aimed to verify the role of this adhesive molecule in EC and especially to check its feasibility as a predictor of lymph node metastases. As already mentioned, CD44 expression, particularly its variant forms (v3, v5, v6, v9), has been analyzed in several malignancies. Increased expression has been attributed to disease progression, metastases and worse prognosis in lymphoma, melanoma, vulvar cancer, cancers of the colon, breast, stomach, ovary, cervix, thyroid and lungs [18–21, 37–39]. On the other hand, loss of CD44v6 correlates with worse prognosis in atypical carcinoids, non-planoepithelial lung cancer, neuroblastoma, and bladder and prostate cancers [18, 20, 21, 39]. This reflects both the complexity of CD44 regulation in cancers, and its tissue specificity [18, 19, 32].

Here, EC cells expressed CD44 more intensively than normal endometrium, in both curettage and surgical specimens (*P* > 0.05 for both), which might reflect CD44′s role in EC, and accords with many other studies [25–31]. The *CD44* gene promoter is activated by the K-ras oncogene product, which also affects splicing of *CD44* mRNA. In 10–37 % of ECs, an activating mutation is reportedly found in codons 12 or 13 of the *K*-*ras* proto-oncogene, and also in 6–16 % of atypical hyperplasia, but not in normal endometrium; this implies that CD44 affects early-stage EC oncogenesis—more in well-differentiated cancers of endometrioid morphology [24, 40]. Its prognostic value is not known.

The CD44 molecule is also physically linked with the product of the *HER*-*2/neu* oncogene —tyrosine kinase and EGF receptor, the expression of which predict poor prognosis in breast and ovarian cancers, and in 9 % of ECs (but in 27 % of metastatic ECs) [7, 28, 40]. Binding of the CD44 with its ligand (HA) stimulates the kinase activity of HER-2/neu and leads to increased proliferation of cancer cells in many tumors, including ovarian cancer [17]. Activation of another tyrosine kinase, c-Src, by the CD44–HA complex stimulates a rearrangement of cytoskeleton proteins and increases migration in breast and ovarian cancers [18, 19, 25]. HA, a primary CD44 ligand and extracellular matrix component can thus promote cancer invasion [25, 41], and may play a similar role in EC where its increased expression, particularly near neoplastic infiltration, correlates with depth of myometrial invasion, low disease grade and LVSI [25, 37, 41]. Its hydrophilic properties help create a semi-liquid environment that facilitates migration and proliferation [19]. However, data on the CD44 expression in endometrium and EC are discordant. Most reports that compare CD44 expression in normal endometrium and in EC found it to be stronger in neoplastic epithelium, as did the present study [25–31]. In healthy endometrium, CD44 is barely expressed, if at all, in the proliferative phase, but is expressed more strongly in the secretory phase [25, 28, 37, 42, 43]. The intensity of reaction increases with the passage from simple and complex hyperplasia to atypical hyperplasia and cancer, and is higher than in normal epithelium irrespective of cycle phase [25, 28, 37], particularly for the CD44v3 and v6 isoforms [25–31]. Saegusa et al. [37] however, found no such difference between simple and complex hyperplasia with or without atypia. HA expression resembles this pattern [25, 37]. However, some data indicate less intensive expression of the CD44 in cancer cells [27, 29, 30, 33].

We observed significantly stronger CD44 expression in resected endometrioid than in papillary serous cancer specimens, where the molecule was virtually absent. This difference was also seen in curettage slides but not significantly so (*P* = 0.15). As papillary serous EC has a worse prognosis, with a stronger tendency to deep myometrial invasion, metastasis formation and relapse, our results could indicate a link between CD44 down-regulation and poor prognosis. However, we had too few patients (5 women) with papillary serous EC to confirm this hypothesis. This disparity in CD44 expression might also reflect differences in pathogenesis of the two EC types, considering the role of K-ras in *CD44* promoter activation in endometrioid EC [24]. Hosford et al. [34] had similar results when they assessed the CD44 expression in papillary-serous cancer: 81 % of specimens expressed no CD44 at all; however they found no significant correlation with prognostic factors.

CD44 expression was weakest in high-grade cancers in resected samples, but did not differ between patients irrespective of grade in curettage specimens, nor were differences significant in any cases. Slightly weaker CD44 expression in high-grade cancers might indicate loss of its function in highly invasive disease. Most other authors did not find a direct correlation between disease grade and CD44, particularly the standard form. Only Ayhan et al. [26] found that poorly differentiated cancers tend to lose CD44v6, which suggests a role for loss of this variant in carcinogenesis. In contrast, Hoshimoto et al. [35] found that the overexpression of CD44v3 significantly correlated with higher grade, similarly to the CD44v6, in the case of which, however, the correlation was not statistically significant. These results suggest that alternative splicing of *CD44* gene transcript may play a role in the EC oncogenesis, especially in poorly differentiated cancers with highly invasive potential—possibly increased use of an alternative splicing mechanism leading to overproduction of variant forms of CD44 in excess of the standard particle. This would explain the lower expression of the CD44 in low-grade cancers in our series. CD44 expression decreased slightly with myometrial invasive depth but the relationship was insignificant. Such a result is consistent with a hypothesis that highly invasive cancers tend to lose the standard variant. A similar but also insignificant relationship of CD44v6 expression was found by Ayhan et al. and Stokes et al. [26, 32], who found that CD44v6 expression strongly correlated with lack of myometrial invasion. Ayhan and Stokes suggest this could be a marker for cancers with myometrial involvement, which would thus facilitate preoperative qualification for lymphadenectomy. It would, however, run contrary to the hypothesis that increased vCD44 expression occurs in more advanced, aggressive tumors. Stokes et al. [32] found standard CD44 (sCD44) expression and depth of invasion to be inversely, but not significantly correlated, which is similar to our findings. On the other hand, Leblanc et al. [28] reported CD44 expression to increase with depth of myometrial invasion. He suggested that alterations of CD44 concentration could mainly be due to local invasion. Such inconsistent results may be due to different methodology and small patient cohorts (Leblanc: 33 patients; Ayhan: 87; Stokes: 40). CD44 expression was not significantly affected by cervical, serosal or adnexal involvement, although in patients with involved serosa or adnexa, CD44 expression tended to be slightly less in both curettage and resected material, which again implies loss of CD44 with more invasive phenotypes. Similar results for CD44v6 were reported by Ayhan et al. [26].

In our group of patients, no significant relationship was seen between CD44 expression and LVSI. In patients with LVSI, expression tended to be higher in curettage specimens and slightly decreased in resection specimens. Such discordance may be due to small cohort size, but may also reflect the fact that curettage is only a blind, random sampling of endometrium, whereas in resected specimens, all sections of the tumor are available. This may be the case as CD44′s expression pattern is locally variable. This could also explain other inconsistencies between pre- and postoperative histological qualification or grading evaluation. In relevant literature, the correlation between CD44 and LVSI also varies. Leblanc et al. and Yorishima et al. [28, 31] found positive relationships between LVSI and CD44v6 expression, which implies that molecules affect local invasion. Stokes et al. [32] reported an opposite tendency for both sCD44 and CD44v6 and other authors–only for CD44v6, which suggests that loss of CD44, particularly CD44v6, is of interest in advanced-stage disease [26, 27, 37, 44]. Other reports showed no relationship between CD44v6 and clinicopathological parameters in EC [36].

In our analysis the CD44 tended to be expressed less in node-positive patients. Although this difference was not significant, this observation could also support the hypothesis that CD44 is lost in more invasive cancers, although other authors’ observations do not concur. Only Yorishima et al. for CD44v6 and Hoshimoto et al. for CD44v3 found positive, significant correlations with node involvement, which suggests dominating roles for variant forms of CD44 in highly invasive tumors [31, 35]. As with other authors, we found no relationship between CD44 and distant metastases or FIGO stage in our data.

## Conclusions

CD44 expression in EC cells fluctuates dramatically, in both preoperative and postoperative specimens: up- or down-regulation, expression of variant forms. In most reports, CD44, and particularly its variants CD44v3 and v6, are expressed significantly more in EC than in normal tissue, especially in early-stage disease. However, CD44 expression tends to decrease as the disease becomes invasive and progressive [26, 27, 37, 44]. Our results tend to support this hypothesis, although without statistical significance. Nevertheless, in some other reports, expression of CD44, CD44v3 and v6 increased with cancer stage [28, 31, 35] or showed no correlation [36].

Altered CD44 expression in pre- and postoperative EC specimens suggests that CD44 affects EC, but not crucially. Lack of both marked differences in CD44 expression in pre- and postoperative analysis and of a direct, straightforward relationship with clinicopathological factors in EC indicate that CD44 is an unfeasible prognostic marker.
